# The Mediation Roles of Coping Modalities on the Relationship Between Stress and Quality of Life Among Jordanian Nurses

**DOI:** 10.1155/2024/4434406

**Published:** 2024-09-30

**Authors:** Wafa'a Ta'an, Yasin Yasin, Mohammed M. Al-Hammouri, Majd Aljabali, Diana Jaradat, Mohammad Suliman, Mohammed Albashtawy, Islam Oweidat, Yazid Al-Hamarneh

**Affiliations:** ^1^ Department of Community and Mental Health Nursing Faculty of Nursing Jordan University of Science and Technology, P.O. Box 3030, Irbid 22110, Jordan; ^2^ College of Health Sciences University of Doha for Science and Technology, Doha, Qatar; ^3^ Princess Salma Faculty of Nursing AL Al-Bayt University, Mafraq, Jordan; ^4^ Community and Mental Health Nursing Department Faculty of Nursing Zarqa University, Zarqa, Jordan; ^5^ Faculty of Medicine and Dentistry University of Alberta, Edmonton, Alberta, Canada

**Keywords:** coping, Jordan, nurse, psychological distress, quality of life, stress

## Abstract

Nurses are at the frontline, dealing with people's most immense healthcare needs in stressful and demanding work environments. Consequently, it is essential to thoroughly examine how various coping mechanisms might affect the relationship between stress and quality of life (QOL). This study aimed to examine the mediation effect of both problem-focused coping (PFC) and emotion-focused coping (EFC) mechanisms on mitigating the effect of stress on the QOL among Jordanian nurses. A multisite cross-sectional descriptive correlational design was used in this study. An online survey was completed by 203 nurses using a convenience sampling technique between October 2023 and January 2024. The study included nurses working in different Jordanian healthcare sectors including governmental, private, and university-affiliated hospitals. Several measures were used to collect data, including questionnaires on sociodemographics, QOL, coping, and stress. Two models were hypothesized for this study. The two models were analyzed using Andrew Hayes Process Macro Model 4 for testing the mediation effects. Additionally, descriptive and correlational analyses were run prior to the main analysis. The results showed that coping significantly mediated the relationship between stress and QOL with variations between PFC and EFC. In conclusion, psychological distress symptoms were common among Jordanian nurses; psychological distress, coping, and QOL are correlating variables. Nurses' stress levels and coping modalities can predict QOL with a superior effect of PFC compared with EFC. Strategies should be put in place to improve effective coping to improve nurses' QOL. The results of this study have important implications for nursing education, practice, future research, and policy.

## 1. Introduction

Nurses are at the frontline to deal with people's most immense healthcare needs. They are, therefore, working in stressful and demanding work environments, requiring attention to nurses' mental health and quality of life (QOL) [1]. Previous studies reported that a high prevalence (81%) of nurses experienced stress mainly due to the hard work nature and being under pressure for extended periods [2]. Similar results were also reported in Jordan highlighting the high prevalence of psychological distress symptoms among Jordanian nurses [3]. Given the scarcity of resources in Jordan, especially the nursing shortage and high turnover, this area stands of foremost importance [4]. The growing shortage in nursing has been a major factor in putting nurses under unrealistic workloads that exceed their abilities and available resources [5]. Work stress has been found to be proportional to the nurses' workload [6]. In other words, as the workload increases, the work stress increases. Consequently, higher levels of stress lead to lower QOL and job performance [7]. Therefore, it is essential to thoroughly examine how various coping mechanisms may influence the relationship between nurses' stress and QOL.

The American Nurses Association launched the Healthy Nurse, Healthy Nation (HNHN) campaign to highlight the importance of improving the health and well-being of nurses and the entire nursing workforce [8]. The campaign focuses on several key areas of wellness, including overall mental health and ways of coping. The overarching aim of this campaign is to be a global initiative that is expandable to developing countries like Jordan. Therefore, as a response to this initiative, studies are needed to shed light on the role of coping mechanisms on nurses' stress and well-being. This study will extend scholarly work to meet and fulfill the ANA goal. Effective coping has been highlighted as a major factor for nurses to overcome psychological distress depending on their risk appraisal [9].

In their seminal work, Lazarus and Folkman [10] proposed that a more efficient coping that matches the person's personal and sociocultural factors can enhance life quality by inducing the “goodness-of-fit” situation. Consequently, coping mechanisms are grouped into two major categories: problem-focused and emotion-focused strategies. According to Lazarus and Folkman, problem-focused coping (PFC) involves cognitive and behavioral strategies to alter or manage the problem causing the stress. Instead, emotion-focused coping (EFC) encompasses managing the emotional response to the stressor without addressing the stressor itself. However, these strategies' usage and effectiveness varied across studies [11, 12]. For example, EFC strategies (EFCSs) were found to be negatively associated with the nurses' psychological distress levels [12], whereas only PFC strategies (PFCSs) were evidenced to reduce stress levels among nurses [13]. Further studies were recommended to thoroughly examine the effect of various coping mechanisms on nurses [14].

The coping strategies that nurses develop and utilize in response to stress are vital, as they substantially impact their QOL [15]. QOL has been defined as “the individual's perception of their position in life in the context of the culture and value systems in which they live and in relation to their goals, expectations, standards, and concerns” [16]. The QOL is considered a complex concept because it encompasses domains related to health, emotional health, and general life satisfaction. Furthermore, it is affected by the outcome of the interaction between the protective factors (e.g., good social support and high socioeconomic status) and the distressing factors (i.e., physical and psychological) [17]. Studies indicated that nurses' QOL is negatively affected by job-related stress [18, 19]. Moreover, nurses' QOL considerably influences their capacity to provide nursing care to their patients [20].

While previous studies have explored the direct relationship between stress and QOL in nurses, few have specifically investigated the coping modalities that mediate this relationship. As discussed above, previous research revealed inconsistencies regarding the impact of EFC versus PFC modalities on nurses' stress levels. Moreover, there is a gap in the literature on this dynamic within the context of Jordanian nurses, who encounter unique challenges shaped by cultural and systemic factors. This study aims to fill this gap by examining how different coping modalities mediate the relationship between stress and QOL among Jordanian nurses. In addition, thoroughly understanding the interactions between stress, various coping strategies, and QOL is essential for promoting nurses' well-being and enhancing healthcare outcomes and may provide valuable insights that can help develop interventions better suited to the needs of Jordanian nurses.

This study focuses on the impact of stress on the QOL among Jordanian nurses, particularly how it is mediated by the coping mechanisms they utilize. This study focuses on this important relationship in Jordan, which is suffering from a shortage in nursing not only due to the demanding health market with a limited supply of qualified professionals but also due to the migration of qualified professionals to other well-paying countries, which exacerbates the problem in shortage in nursing and increased work demand and associated consequences. For example, Jordan has been witnessing a huge migration of qualified nurses to the Gulf Cooperation Council (GCC) states since 1990 [21].

**Figure 1:**
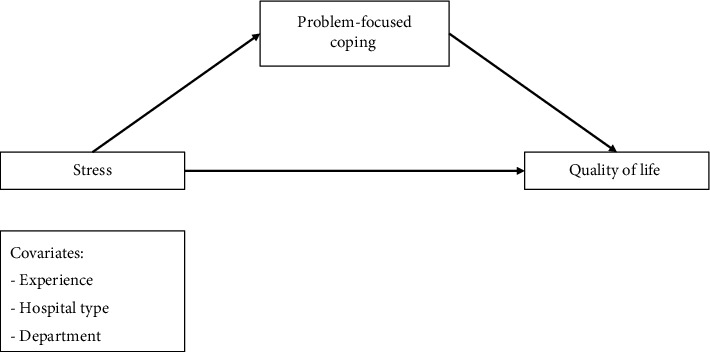
The conceptual framework for problem-focused coping as a mediator of stress–QOL relationship (Model 1).

**Figure 2:**
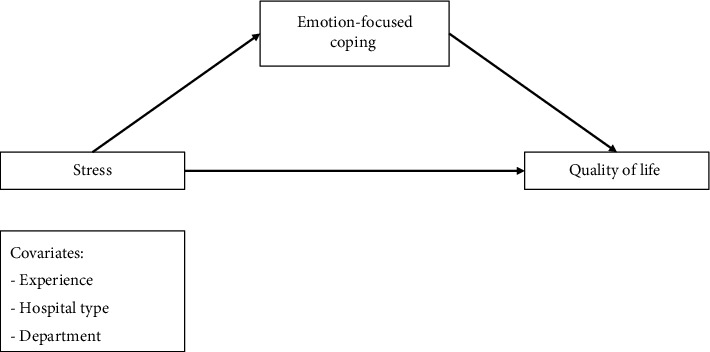
The conceptual framework for emotion-focused coping as a mediator of stress–QOL relationship (Model 2).

This study aims to explore the differential effects of PFCSs versus EFCSs on mitigating the adverse effects of stress on Jordanian nurses' QOL. We proposed two conceptual models, illustrated in Figures 1 and 2. The first model suggests that stress indirectly impacts nurses' QOL through PFC, proposing a mediation relationship where PFC serves as the intermediary. Conversely, the second model posits EFC as the mediating factor, hypothesizing that it influences the stress–QOL relationship.

The specific objectives are to describe stress, coping strategies, and QOL among nurses in Jordan and to test the mediation effect of two coping mechanisms (PFC and EFC) on mitigating the effect of stress on QOL among nurses in Jordan. Understanding the magnitude of these variables and their interplay is crucial for enhancing the overall quality of nursing care and promoting nurses' well-being. This research seeks to provide insights into the mechanisms in which the study variables interact among Jordanian nurses and contribute to the development of tailored interventions that can promote their well-being and enhance healthcare outcomes.

## 2. Materials and Methods

A cross-sectional multisite correlational design employing an online survey was used in this study to investigate the relationship between the study variables. The study was conducted in healthcare settings from different Jordanian healthcare sectors including governmental, private, and university-affiliated hospitals with a bed capacity of 100 or more to ensure that the nursing workforce care achieves the needed sample. Accredited hospitals were included; this criterion was incorporated to ensure the presence of a minimal level of standardized nursing care and leadership supervision.

The sample size was calculated using Monte Carlo power analysis for indirect effects. The sample size was calculated for the indirect effect model with one mediator, which is the one used in the current study. This calculation required the determination of the set values, including the standardized coefficients of the association between the independent variable and the mediator (a), the mediator and the dependent variable (b), and the independent variable and the dependent variable (c). These values were obtained from Dardas and Ahmad [22], which holds the closest similarities to the current study. Dardas and Ahmad [22] studied the same combination of variables and sets of relationships but with different populations. The parameters from their study we used to calculate the sample size required in the current study were *a* = 0.320, *b* = −0.236, and *c* = −0.05. Based on these parameters, the minimum sample size to achieve a power of 0.8 (LLCI: 0.77 and ULCI: 0.83) was 160 participants.

An online questionnaire was used. The questionnaire consisted of four parts: the participants' sociodemographic questionnaire; the revised ways of coping questionnaire (RWCQ) developed by Lazarus and Folkman [23]; the Depression, Anxiety and Stress Scale—21 Items (DASS-21) developed by Lovibond and Lovibond [24]; and the EUROHIS-QOL index developed by Schmidt et al. [25].

The DASS-21 is a questionnaire developed by Lovibond and Lovibond [24] to measure psychological distress symptoms of depression, anxiety, and stress. The questionnaire consists of three subscales containing seven items each. Each item rates as follows: (0) did not apply to me at all—never; (1) applied to me to some degree, or some of the time—sometimes; (2) applied to me to a considerable degree, or a good part of the time—often; and (3) applied to me very much, or most of the time—almost always. To calculate the final subscale total scores for the DASS-21, the total scores must be multiplied by 2. The classification of the stress subscale is based on the following ranges: normal: 0–10, mild: 11–18, moderate: 19–26, severe: 27–34, and extremely severe: 35–42. The Arabic version was validated in [26], and it was used in this study. The Cronbach alpha value of the stress subscale in this study was 0.81 representing very good reliability.

To measure the QOL, the EUROHIS-QOL eight-item index was used, which is a shorter, more practical, faster, and easier-to-administer tool compared to the original WHOQOL versions [25]. The original EUROHIS-QOL index was developed from the WHOQOL project to measure QOL internationally [27]. The EUROHIS-QOL is a unidimensional tool that consists of eight items that can be rated on a five-point Likert scale ranging from “1: not at all” to “5: completely.” The EUROHIS-QOL eight-item index was psychometrically tested and demonstrated acceptable cross-cultural performance and adequate discriminant validity [28]. The Cronbach alpha of the Arabic version in this study was 0.88 representing very good reliability.

The RWCQ was developed by Folkman and Lazarus [23]. The tool is a self-report measure that contains 50 items and consists of two coping categories with four subscales for each. The first is PFCSs with 24 items that include the following subscales: self-controlling (7 items), accepting responsibility (4 items), planful problem-solving (6 items), and positive reappraisal (7 items). The second is EFCSs with 26 items compromising confrontive coping (6 items), distancing (6 items), seeking social support (6 items), and escape avoidance (8 items) subscales. Each item is rated on a 0 to 3 Likert scale with 0 representing “not used” to 3 for “used a great deal.” The scale was psychometrically tested and found to be valid and reliable (*α* = 0.77–0.90) [29]. The Arabic version of the survey was unavailable, so we adhered to the World Health Organization's guidelines for questionnaire translation. The Cronbach alpha values of the eight subscales of the RWCQ in this study ranged from 0.67 to 0.83. The PFCSs and EFCSs were also tested for internal consistency in this study, and the Cronbach alpha values were 0.93 and 0.92, respectively.

The data were collected during the period from October 2023 to January 2024 from nurses working in three hospitals in Jordan. The researchers contacted nursing administrators to encourage nurses to participate in the study during their official channels of communication within each healthcare institution. Nurses are eligible to participate if they are registered nurses and have at least 1 year of experience. The data collection procedure took place by distributing online self-administered questionnaires to potential participants who met the inclusion criteria through professional social network websites and WhatsApp groups. A link that directs potential participants to the questionnaire was posted, leading to the letter of information, the inclusion criteria, and an estimated time to complete the survey. Prospective participants were instructed to fill out the questionnaire only once. By clicking agree to participate, the online questionnaire opens. A message appears at the end to thank participants for completing the questionnaire.

The Statistical Package for the Social Sciences (SPSS) version 27 with the Process v 4.0 by Andrew F. Hayes plug-in was used for data analysis. Before analysis, data were screened for missing and unengaged responses. A total of 12 responses were excluded because they missed more than 10% of the items resulting in a final sample of 203 participants. Normality, skewness, and kurtosis were also assessed using descriptive statistics on SPSS before proceeding to parametric tests. This was done along with other assumptions tests to ensure that the dataset meets the regression analysis assumptions. The mean and standard deviation (SD) were used to describe the continuous variables. In addition, frequencies and percentages were used to describe dichotomous variables.

In addition, the Andrew Hayes Process Macro Model 4 was used to conduct the mediation analysis to test the conceptual models, suggesting that stress affects the QOL in several direct and indirect pathways by using PFC versus EFC mechanisms as illustrated in Figures 1 and 2. Demographic variables of age, hospital type, and department were entered into the model as possible covariates. In the mediation analysis, a confidence interval of 95% and 5000 bootstrap resamples were considered to estimate the results' significance. As per Hayes' [30] recommendation, the analysis results were judged significant when “zero” did not fall in the range between the upper and lower limits of the 95% confidence interval. The significance level was set to a *p* value of less than 0.05. In our study, as the dependent and independent variables were measured using a single survey using the same response method, we used Harman's single-factor test to check whether we had a common method bias. To achieve that, we used the exploratory factor analysis test (unrotated solution). This method is the most widely used method to detect common method bias [31].

The research approval was obtained from the Institutional Review Board at the principal investigator's affiliated university. Potential participants were invited to participate in the study. They were given the freedom to participate or withdraw anytime. The study's objectives were fully described to participants before participation. To ensure participants' anonymity and confidentiality, coding of the questionnaires was done, row data were destroyed, and only anonymous data were kept for the data analysis. The participants were assured that the results would be shared and used for research purposes only.

## 3. Results

### 3.1. Sample Characteristics

The study questionnaire was completed by a total of 203 nurses, comprising 35 (17.2%) males and 168 (82.8%) females. The respondents were employed across various settings: 82 nurses (40.4%) worked in governmental hospitals, 76 (37.4%) in university-affiliated hospitals, and 45 (22.2%) in private hospitals. The participants' average age was 31.3 years (SD = 5.9), with a mean of 8.1 years (SD = 5.7) of experience in their current roles. The majority of participants held a bachelor's degree (*n* = 146, 71.9%), whereas 35 (17.2%) possessed a master's degree and 22 (10.8%) had a nursing diploma.  Table 1 provides a comprehensive overview of the participant's demographic characteristics. Harman's single-factor test produced a one-factor solution that accounted for 27.07% of the variance, indicating the absence of the common method bias, as the explained variance was below the cutoff value of 50% [32].

### 3.2. Description of Stress, Coping, and QOL

In this study, nurses reported a moderate overall QOL (mean = 25.6, SD = 15.64). The majority of the nurses who participated in this study (65%) reported a moderate QOL level, whereas 17% had a low QOL level and only 18% had a high QOL level. The DASS-21 scores indicate that the mean stress score was 23.3 (SD = 6.73), which can be classified as moderate (19–26) (Table 2).

Regarding the RWCQ, the PFCSs had a subtotal mean of 29.9 (SD = 6.8), whereas the EFCSs had a subtotal mean of 42.5 (SD = 9.8). This indicates that the participants used EFCSs more frequently compared with PFCSs. The PFCSs encompassed self-controlling with a mean of 7.03 (SD = 7.0), accepting responsibility with a mean of 5.27 (SD = 5.3), planful problem-solving with a mean of 8.94 (SD = 8.9), and positive reappraisal with a mean of 8.63 (SD = 8.6). Among these, planful problem-solving was the most utilized strategy. The EFCSs had a subtotal mean of 42.5 (SD = 9.8), and the individual EFCSs included confrontive coping with a mean of 10.83 (SD = 2.6), distancing with a mean of 10.22 (SD = 2.3), seeking social support with a mean of 10.82 (SD = 2.8), and escape avoidance with a mean of 10.67 (SD = 3.0). In the EFCSs category, confrontive coping was the most frequently employed strategy.

### 3.3. Mediation Analyses

To investigate the mechanisms by which PFC versus EFC mechanisms work conditions mitigate nurses' stress effect on their QOL, the study's conceptual models were examined using Andrew Hayes Process Macro Model 4. The QOL was inserted as the outcome variable, stress was the independent variable, and PFC and EFC mechanisms were the mediators. The analysis showed that Model 1 (PFC as mediator) was significant (*F* [5,197] = 6.64, *p* < 0.001) predicting 14% of the variance in nurses' QOL. Model 2 (EFC as mediator) was also significant (F [5,197] = 5.70, *p* < 0.001) predicting 13% of the variance in nurses' QOL. The mediation analysis results, summarized in Table 3, demonstrated that both direct and indirect effects were significant resulting in partial mediation of both PFC and EFC. By examining the effect direction, it was concluded that the mediation was competitive, not complementary.

The coefficient analyses (Table 4) showed that only stress and coping were significantly correlated with QOL, whereas the covariates (experience, hospital type, and unit) were not significantly correlated with QOL. To shed light on the variation among the coping mechanisms, the coefficient of the PFC was higher than that of the EFC (beta = 0.20, *p*  <  0.01; beta = 0.12, *p*  <  0.01 for PFC and EFC, respectively).

## 4. Discussion

The findings of this study indicated that participants experienced a moderate level of stress. This result is alarming as psychological distress symptoms have been documented to negatively affect the individual”s well-being and quality of patient care [33]. Consistently, Lai et al. [34] reported that 71% of healthcare workers were distressed with nurses having higher stress scores compared with other healthcare professionals.

In the current study, the QOL for Jordanian nurses is found to be moderate. This finding is congruent with a previous study that reported good to average QOL scores among nurses [35]. Regarding coping modalities, Jordanian nurses used EFCSs more frequently compared to PFCSs, with planful problem-solving and confrontive coping being the most common within their respective categories. Such results indicate nurses' tendency to use strategies that are focused on the external environment rather than changing their own behavior or thought patterns. Consistently, a previous study conducted in Singapore revealed that nurses' most commonly used strategies were seeking social support, escape avoidance, and positive reappraisal to cope with stress [36].

This study focused on comparing PFCSs and EFCSs on the relationships between stress and QOL. However, future research is warranted to examine the specific work situations or environmental factors that may trigger the use of one strategy over another. Situational and environmental factors, such as workload intensity, limited resources, staffing shortages, and interpersonal conflicts, may influence the choice of coping strategy. Future research could explore these factors in more detail to better understand how nurses decide between PFC and EFC based on the unique challenges they encounter in their work environment.

The mediation analysis provides insightful results on the role of coping modalities (PFC and EFC) in mitigating the risk of stress on nurses' QOL. The analysis concluded the presence of competitive partial mediation in both models. In particular, there is a significant negative direct effect of stress on QOL, indicating that higher stress levels are associated with lower QOL, which is consistent with the findings of other worldwide studies [3, 37, 38]. For example, Alhawatmeh et al. [37] studied the relationship between stress and QOL among 550 Jordanian nurses, and the results of their study revealed a direct negative relationship between stress and QOL (*β* = −0.34, *p*  <  .01). Sarafis et al. [38] also studied these variables among 246 nurses in Greece and found a significant negative correlation between stress and QOL.

However, the indirect effect, mediated through PFC, is positive, indicating that PFC mitigates the negative impact of stress on QOL with a significant coefficient of 0.20, whereas EFC had a significant coefficient of only 0.12. This suggests that PFC, which involves tackling the stressors directly, is more efficient than EFC, which involves managing emotional responses, in improving the overall QOL for nurses. The findings of this study were consistent with a previous study by Martínez-Zaragoza et al. [39], which examined how nurses use problem- and emotion-focused strategies to cope with daily stressors. The study found that PFC was reported to have a positive impact on handling stress, leading to positive impacts. However, EFC was less effective in managing specific tasks and more influenced by the overall emotional and physical state of the nurse, such as mood and fatigue levels. In conclusion, while EFC can help mitigate the immediate emotional impact of stress, PFC demonstrated a superior effect in reducing long-term stress levels compared with PFC.

The results of this study suggest that employing effective coping strategies can help nurses better manage the challenges and pressures associated with nursing, ultimately leading to an enhanced QOL. Conversely, stress was observed to have a negative association with QOL. This highlights the importance of addressing and mitigating stressors to promote better QOL outcomes for nurses. Finally, the study concludes that PFC is more effective in mitigating the risk of stress on nurses' QOL requiring nurse managers to invest in creating strategies targeting enhancing PFC among nurses to improve their well-being and eventually the patient quality outcomes.

Nursing curricula should emphasize the long-lasting effects of psychological stressors that might be triggered by work overload and include an understanding of how nurses' coping strategies are critically important to adapt to stress. Where possible, clinical simulations are needed when working in high-demand situations to provide key prospects to incorporate theory into practice. For research, additional studies are needed to assess QOL among nurses who have experienced stressful situations and investigate other variables that may contribute to a better understanding of this area.

This study recommends establishing policies and making decisions to provide PFC training and psychosocial support services for nurses such as professional development programs that can help nurses develop a balanced approach to stress management. These programs may include training on planning, time management, and seeking solutions to problems to enhance their PFCSs. In addition, workshops and interactive activities about safety measures during challenging situations may promote positive outcomes for healthcare professionals and health organizations.

## 5. Limitations and Delimitations

Although these study results add valuable findings to the nursing literature, we acknowledge some limitations. The main limitation of this study is the use of cross-sectional data for mediation analysis, which limits our ability to establish causality and assess the temporal dynamics among variables. Cross-sectional mediation can result in biased estimates, particularly for processes that unfold over time [40]. While our analysis is theoretically sound, the cross-sectional design requires cautious interpretation, with findings considered preliminary. Validation through longitudinal studies is needed to confirm the proposed mediation effects. Despite this, our study provides valuable insights by capturing nurses' psychological distress, coping, and QOL, offering new perspectives on factors influencing nurses' well-being. The diverse sample—covering various ages, experience levels, genders, and work settings—enhances the generalizability of the results.

The RWCQ was not previously available in Arabic. Therefore, the current study used a standard translation procedure and tested the translated measure's internal consistency. Future psychometric studies on the translated version of the questionnaire are warranted. The results of this study provide important evidence of the relationships between the main study variables among Jordanian nurses, which can be further investigated and compared with professional or work-related QOL.

## 6. Conclusions

To our knowledge, this was the first study to examine the mediating role of coping modalities on the relationship between stress and QOL among nurses in Jordan. Results from this study indicate that a positive, significant correlation exists between coping and QOL, whereas a negative, significant correlation exists between QOL and stress. This study also provides evidence that PFC, compared to EFC, has a higher effect in reducing the effect of stress on nurses' QOL. The results of this study have important implications for nursing education, practice, future research, and policy.

Nurses' reactions to stress produced by challenging work circumstances must be seen from safety and occupational health perspectives. The findings emphasize the importance of promoting PFCSs among nurses to enhance their ability to deal with stress effectively and improve their QOL. Training and support systems could be oriented more toward problem-solving skills to leverage this coping mechanism's beneficial effects. The results of this study suggest that strategies aiming to enhance nurses' control over their workplace are important. Nurses need to be encouraged to evaluate their QOL over time, which can contribute to improving the quality of the care they provide. Enhancing coping strategies programs should be given serious attention by healthcare agencies. Reaching out to nurses who have experienced distress to evaluate their QOL would be an important component of a long-term secondary prevention strategy [41].

## Figures and Tables

**Table 1:** Demographic characteristics of participants (*n* = 203).

**Variable**	**Range**	**Mean**	**Standard deviation**
Age	21–46	31.32	5.93
Experience	1–32	8.06	5.66

	**Categories**	**Number**	**Frequency (%)**

Gender	Female	168	82.8
	Male	35	17.2

Marital status	Married	123	60.6
	Single	76	37.4
	Divorced	3	1.5
	Widowed	1	0.5

Educational level	Diploma	22	10.8
	Bachelor's degree	146	71.9
	Master's degree	35	17.2

Hospital type	Governmental	82	40.4
	University	76	37.4
	Private	45	22.2

Department	Critical unit	32	15.7
	Floor	127	62.6
	Emergency	24	11.8
	Other (dialysis, operation, clinic)	20	9.9

**Table 2:** Descriptive statistics and Cronbach's alpha levels for the study variables (*n* = 203).

**Variable**	**Min**	**Max**	**Mean ± SD**	**Cronbach's alpha**
*Stress*	14	42	23.3 ± 6.73	0.81
*RWCQ*				
*PFCSs (subtotal)*	17	49	29.9 ± 6.8	0.93
Self-controlling	4	12	7.03 ± 7.0	0.78
Accepting responsibility	3	9	5.27 ± 5.3	0.60
Planful problem-solving	5	15	8.94 ± 8.9	0.80
Positive reappraisal	5	15	8.63 ± 8.6	0.83
*EFCSs (subtotal)*	24	70	42.5 ± 9.8	0.92
Confrontive coping	6	18	1.83 ± 2.6	0.67
Distancing	6	18	10.22 ± 2.3	0.79
Seeking social support	6	18	10.82 ± 2.8	0.77
Escape avoidance	6	18	10.67 ± 3.0	0.78
*QOL*	8	40	25.6 ± 15.64	0.88

Abbreviations: EFC, emotion-focused coping; PFC, problem-focused coping; QOL, quality of life; RWCQ, revised ways of coping.

**Table 3:** Mediation analysis summary.

**Relationship**	**Total effect**	**Direct effect**	**Indirect effect**	**Confidence interval**		**t-statistics**	**Conclusion**
				**Lower bound**	**Upper bound**		
Stress–PFC–QOL (Model 1)	−0.28 (*p* < 0.01)	−0.31 (*p* < 0.01)	0.04	0.01	0.08	−4.33^∗∗^	Competitive partial mediation
Stress–EFC–QOL (Model 2)	−0.28 (*p* < 0.01)	−0.31 (*p* < 0.01)	0.03	0.003	0.073	−4.33^∗∗^	Competitive partial mediation

Abbreviations: EFC, emotion-focused coping; PFC, problem-focused coping; QOL, quality of life.

^∗∗^ *p* < 0.01.

**Table 4:** Regression coefficient analysis.

**Variable**	**Unstandardized coefficients**		**Std. beta**	**t**	**Sig.**
	**Beta**	**Std. error**			
*Problem-focused coping model (Model 1)*					
Stress	−0.31	0.06	−0.33	−4.97	<0.01
PFC	0.20	0.06	0.24	3.54	<0.01
Experience	<0.01	0.07	<0.01	−0.01	0.99
Hospital type	0.20	0.48	0.03	0.41	0.67
Unit	0.39	0.52	0.05	0.75	0.45

*Emotion-focused coping model (Model 2)*					
Stress	−0.30	0.06	−0.33	−4.82	<0.01
PFC	0.12	0.04	0.19	2.87	<0.01
Experience	<0.01	0.07	<0.01	−0.03	0.98
Hospital type	0.16	0.49	0.03	0.33	0.73
Unit	0.37	0.53	0.05	0.70	0.48

*Note:* QOL is the dependent variable.

Abbreviations: EFC, emotion-focused coping; PFC, problem-focused coping; QOL, quality of life.

## Data Availability

The study dataset is available upon reasonable request from the corresponding author with permission from Jordan University of Science and Technology.
